# Two pursuit strategies for a single sensorimotor control task in blowfly

**DOI:** 10.1038/s41598-020-77607-9

**Published:** 2020-11-27

**Authors:** Leandre Varennes, Holger G. Krapp, Stephane Viollet

**Affiliations:** 1 Aix Marseille Univ, CNRS, ISM, Marseille, France; 2 Department of Bioengineering, Imperial College, London, SW7 2AS UK

**Keywords:** Biophysical models, Dynamical systems, Aerospace engineering

## Abstract

Effective visuomotor coordination is a necessary requirement for the survival of many terrestrial, aquatic, and aerial animal species. We studied the kinematics of aerial pursuit in the blowfly *Lucilia sericata* using an actuated dummy as target for freely flying males. We found that the flies perform target tracking in the horizontal plane and target interception in the vertical plane. Our behavioural data suggest that the flies’ trajectory changes are a controlled combination of target heading angle and of the rate of change of the bearing angle. We implemented control laws in kinematic models and found that the contributions of proportional navigation strategy are negligible. We concluded that the difference between horizontal and vertical control relates to the difference in target heading angle the fly keeps constant: 0° in azimuth and 23° in elevation. Our work suggests that male *Lucilia* control both horizontal and vertical steerings by employing proportional controllers to the error angles. In horizontal plane, this controller operates at time delays as small as 10 ms, the fastest steering response observed in any flying animal, so far.

## Introduction

In-flight capture is considered one of the fastest behaviours in the animal world. Some predators catch their food on the wing like eagles^1^, falcons^2^ and bats^3^. Invertebrates such as dragonflies are fine aerial hunters with capture success rates up to $97\%$^4^. The fastest trajectory adjustments in the range of 20 ms observed so far were reported for male dipteran flies when pursuing a female conspecific on the wing^5^. In the event of predation or reproduction, the survival of these species depends on the successful capture of the target. With the massive development of robotics, it became possible to reconstruct some insect behaviors^6^ such as exploring and returning home^7^, following a wall^8,9^, landing on target^10^ and avoiding obstacles^11,12^. But in aerial pursuit, the robots’ performances are far from aerobatics of real insects^13^. To replicate a pursuit behavior found in nature, it becomes mandatory to investigate the animal’s sensorimotor control laws.

Taking advantage to the emergence of high-speed videography in the 1970s, Land and Collett carried out the first experiments to study aerial tracking on the housefly *Fannia* sp.^14^. Based on their free flight data, they developed a kinematic model formally described as a proportional derivative, PD, controller with proportional and derivative gains (*kp* and *kd*, respectively), including a time delay ($\Delta t$). This was followed by studies on other species such as hoverfly^15^, housefly^16^ and blowfly^17^. In several cases the different pursuit strategies across species were correlated with specific anatomical and neuronal adaptations supporting the behaviour^18,19^.

**Figure 1:**
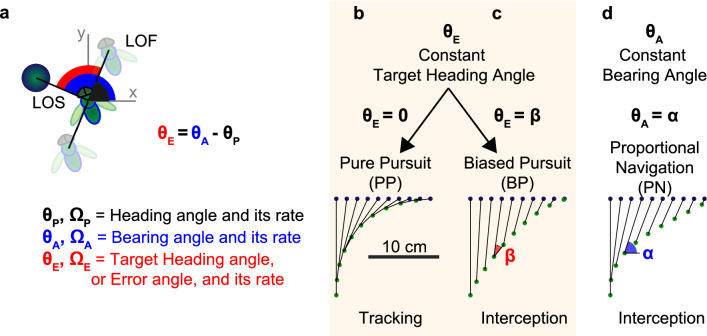
Definition of angular parameters during pursuit, and planar pursuer’s trajectories with different steering controls. (**a**) Plan view of angular parameters during pursuit. *x* and *y*-axes form an external frame of reference, Line of flight, *LOF*, connects successive positions of the pursuer, and line of sight, *LOS*, connects the pursuer to the target. Pursuer’s heading angle, $\theta _P$, is formed between *LOF* and *x*-axis, bearing angle, $\theta _A$, between *LOS* and *x*-xis, and target heading angle, $\theta _E$, is the difference between $\theta _A$ and $\theta _P$. (**b**–**d**) Simulation of different pursuit strategies. Steering controllers are divided in two categories. First category aims to maintain a constant target heading angle $\theta _E$, (**b**) to zero in the case of pure pursuit, *PP*, or (**c**) to a non-zero angle, $\beta$, in the case of biased pursuit, *BP*. The other control category maintains a constant bearing angle $\theta _A$ to a non-zero angle, $\alpha$, and is presented in (**d**) by proportional navigation, *PN*. For pursuit simulations, the target linear speed is 1 m/s and the pursuer’s speed is 1.5 m/s. The positions of target and pursuer (dark blue and green, respectively) are shown every 20 ms. *LOS* is shown in black. (**b**) *PP* with $kp = 1\;\text{s}^{-1}$ and $\Delta t = 0\;\text{s}$ leading to a tracking strategy. (**c**) *BP* with $kp = 1\;\text{s}^{-1}$ , $\Delta t = 0\;\;\text{s}$ and ‘bias angle’ $\beta = -30$°, leading to an interception. (**d**) *PN* with $N = 3$ and $\Delta t = 0\;\text{s}$, leading also to an interception.

For a capture to take place the pursuer and the target have to be in the same place at the same time. Before this can happen, the pursuer must continuously maneuver according to the movements of the target. This is the sensorimotor control task. The controller takes as visual input an angular parameter between pursuer and target, and by series of basic neuronal operations, and muscular action it adjusts the steering—i.e changing heading—to stabilize the angular input. Two angles link together the pursuer and the target: one in the pursuer reference frame, the target heading angle, $\theta _E$, and one relative to an external frame of reference, the bearing angle, $\theta _A$. Relationship between pursuer’s heading angle, $\theta _P$, and the target’s relative angles, $\theta _E$ and $\theta _A$, are presented in Fig. 1a. Angular definitions in pursuit literature may differ between research groups, however in this study we will follow notation used in human ecology, where bearing is defined with respect to an exocentric (allocentric) frame of reference^20–22^. In this section we will present pursuit strategies that rely on stabilizing $\theta _E$, $\theta _A$, or both (equations are given in Table 1).

The mathematical tools proposed to study chases and escapes date back to antiquity. They advanced during the Renaissance with the boom in maritime trade and the problems of piracy. A famous pursuit problem, ‘dog tail’ or classical pursuit, was described by Pierre Bouguer, a French mathematician and hydrographer in a paper published in the French Academy’s *Memoires de l’academie royale des sciences* in 1735 (from^23^). It presents the trajectory of a pursuer in the case where the pursuer aligns its velocity vector towards the current position of the target, this way the pursuer stays in the wake of the target. This strategy was later observed in tiger beetles^24^, houseflies^14^, blow flies^17^, and honey bees^25^ (Fig. 1b), and is now referred as pure pursuit, *PP*. The *PP* control can be described by a simple gain—proportional term—as described in Eq. (1.1), or a proportional and a derivative term, applied to the target heading angle, $\theta _E$. This controller aims at stabilizing $\theta _E$ to zero.

**Table 1:** Equations governing steering for different pursuit strategies.

Control law for steering	Equation	
Constant target heading angle (*CTHA*)	$\displaystyle \Omega _P (t) = f(\theta _E (t))$	
Pure pursuit (*PP*)	$\displaystyle \Omega _P (t) = kp \cdot [\theta _E (t - \Delta t) + \beta ]$ , with $\beta = 0$	(1.1)
Biased pursuit (*BP*)	$\displaystyle \Omega _P (t) = kp \cdot [\theta _E (t - \Delta t) + \beta ]$ , with $\beta = constant$	(1.2)
Constant bearing angle (*CBA*)	$\displaystyle \Omega _P (t) = g(\theta _A (t))$	
Proportional navigation (*PN*)	$\displaystyle \Omega _P (t) = N \cdot \Omega _A (t - \Delta t)$	(1.3)
Hybrid control (*CTHA* + *CBA*)	$\displaystyle \Omega _P (t) = f(\theta _E (t)) + g(\theta _A (t))$	
Mixed pursuit (*MP*)	$\displaystyle \Omega _P (t) = kp . [\theta _E (t - \Delta t_1) + \beta ]$ $+ N . \Omega _A (t - \Delta t_2)$	(1.4)

The controller can use two angles as input: target heading angle ($\theta _E$) or bearing angle ($\theta _A$), and will stabilize it while changing the pursuer heading by mean of functions *f* in *CTHA*, and *g* in *CBA*. For pure pursuit and biased pursuit, *f* is a first order function, with gain, *kp* and time delay $\Delta t$. Proportional navigation is a first order function (*g*) with gain *N*, and time delay $\Delta t$, applyed on first temporal derivative of the bearing angle. Mixed pursuit is addition of the two controllers *BP* and *PN*.

While during tracking the chaser is heading towards the target’s position, during interception it aims at a point in front of the target. Classical interception, also called deviated pursuit strategy in the interception literature, aims to maintain a constant (but non-zero) target heading angle, which we call here bias angle $\beta$ (see Eq. (1.2) and trajectory in Fig. 1c). The term ’deviated’ describes a temporary event, whereas in the technical literature ’non-zero error’ mostly refers to an offset angle. We therefore introduce the term ’biased’ when referring to a pursuit strategy that keeps the target at a constant, non-zero angle. In hoverflies *Eristalis* and *Volucella*, males use their innate knowledge of female’s size to compute the optimum interception angle based on the combination of position and angular speed of the target^26^. Other species maintain the bias angle constant throughout the pursuits such as Bluefish *Pomatomus saltatrix*, who keeps a 10° horizontal bias angle^27^. Dragonflies use a biased pursuit strategy in the vertical plane to hold the target image in the dorsal acute zone, a crescent of a particularly high resolution about 55° above the eye equator. Behavioural experiments in dragonfly have shown that the pursuer keeps the target in this region when hunting flying-insect prey^28^. The dorsal acute zone in the dragonfly *Sympetrum* is exclusively sensitive to short wavelengths of light (blue and UV)^29^, a regional specialization for foraging against the blue sky. In their acute zones some dragonfly species feature a remarkably high spatial resolution in the range of about 0.1°, which is—apart from some robberflies^30^—probably the best found in any insect/arthropod species.

Steering controls that aim to maintain the target heading angle constant can thus lead to different pursuit strategies. When the system stabilize the target heading angle to zero the pursuer present a tracking strategy, and when it stabilize to a non zero constant, the pursuer follow an interception path.

The other control category maintains a constant bearing angle $\theta _A$ (Fig. 1d). Proportional navigation, PN, is often used in the aerospace industry for missile guidance^31^ as it was considered as a control strategy with energy saving optimum^32^. An image to exemplify the situation is that of a pursuer shadowing a prey from an infinite distance away. The change of course is governed by changes of the bearing angle multiplied by a factor, *N*, between 1 and 5, see Eq. (1.3). This control strategy has been found in an insectivorous echolocating bat^33^, killer fly and robber fly^30,34^. The latest comparative study^1^ suggests that a small *N* is more effective in cluttered environments and with highly-manoeuvrable targets (see killer fly with $N=1.5$^30^). If $N = 1$, *PN* is similar to *PP*, and assures a capture in any case, if the pursuer’s speed is higher than that of the target. If *N* gets higher (3–5), the pursuer will perform a parallel navigation path, also called *Constant Absolute Target Direction* strategy^33^, which is optimal for low-manoeuvrable target, or for high-speed chasers operating in open field such as peregrine falcon^2^ and some robber flies^30^. In practice, it is not very clear how the animal measures this absolute bearing angle to keep it constant. An idea could be the addition of $\theta _E$ and $\theta _P$ (Fig. 1a), but it supposes animal can estimate it’s own orientation. The fly could also use first temporal derivatives, since changes in body orientation may be sensed by the fly’s gyroscopic halteres which measure body rotation rates^35^, and changes in error angle encoded in male specific visual neuron MLG1^36^.

Brighton and Taylor^1^ first showed the possibility of a mixed orientation law in hawk adding *PP* and *PN* (Eq. (1.4)), that would give an advantage when the target moves fast or in a cluttered environment. This strategy has been used in missile guidance^32^.

Our work aimed to identify the control strategies underlying aerial pursuit in the male blowfly *Lucilia sericata*. To this end, we carried out a series of experiments in which male flies were chasing dummy females moving on a computer-controlled 2d trajectory. The resulting 3-dimensional free flight data enabled us to study strategies the flies apply to control their steering in the horizontal and the vertical planes.

## Results

Olberg et al.^4^ proposed a static approach to define the pursuit strategy of the dragonfly. The authors compared the variations of $\theta _E$ and $\theta _A$ during pursuits. They discovered an average variation of 2.8° for the bearing angle $\theta _A$, and 8° for the error angle $\theta _E$. As the variation is smaller for the bearing angle, the authors proposed that the dragonfly changes course in order to keep $\theta _A$ constant. Based on our experimental data we argue that the study of the distribution of $\theta _E$, $\theta _A$ and $\theta _P$ gives important information but it will be necessary to perform a thorough temporal analysis of the trajectories to derive a robust control system. To propose a 3D kinematic model of the pursuit behaviour, we analysed thoroughly the angular distribution of the main angles defined in Fig. 1. We also achieved cross correlation between angles and their rates. Finally, we analysed the flight speed of the flies.

### Distribution of invariant parameters

#### In azimuth

**Figure 2:**
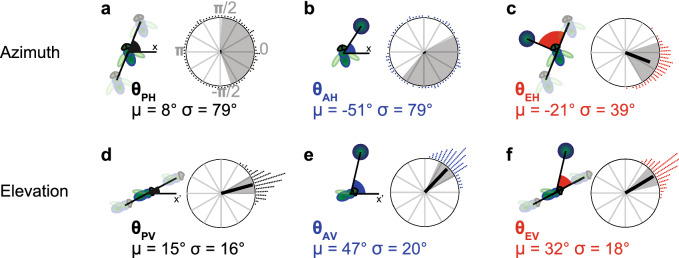
Distribution of the angular parameters: mean vector and standard deviation (**a**–**c**) in the horizontal plane, and (**d**–**f**) in the vertical plane. The mean vector, thick black line, points to the direction of the mean angle, and its length is linked to the data distribution: 0 if uniformly distributed, or 1 (radius) if all data are centred on a single value. In (**a**,**b**), horizontal heading angle $\theta _{PH}$ and horizontal absolute bearing angle $\theta _{AH}$ are uniformly distributed, and mean vector is barely visible. In (**c**–**f**) horizontal bearing angle $\theta _{EH}$ and all vertical angles $\theta _{PV}$ , $\theta _{AV}$ and $\theta _{EV}$ are centred on specific values with small variation. $\mu$ is the angular mean and $\sigma$ the angular standard deviation. Data were gathered by 5° steps, each dot represents 10 measures (N = 1100). Shaded areas indicate $\mu \,{\pm } \, \sigma$.

Pursuer heading angle $\theta _{PH}$ and bearing angle $\theta _{AH}$ (*P* for pursuer, *A* for absolute bearing angle, and *H* for horizontal plane) are uniformly distributed, making their mean vectors’ length almost equal to zero (Fig. 2a,b). In other words, the pursuer flies and chases in any direction. The mean vector of the target heading angle, $\theta _{EH}$ was centred on − 21° (Fig. 2c). The preferred direction angle − 21° is an offset due to the definition of the direction of rotation of the target (see^37^). The length of its mean vector suggests that in the horizontal plane, the fly is using a constant target heading angle controller. On the other hand, because of the large variance of $\theta _{AH}$ this angle is unlikely to be used for the controls within the horizontal plane, which excludes the constant bearing angle controller and thus the proportional navigation strategy, *PN*.

#### In the vertical plane

$\theta _{PV}$ and $\theta _{AV}$ differ in their mean value, 15 and 47°, respectively, but they both show small standard deviation, 16 and 20°, respectively (Fig. 2d,e). The vertical error angle $\theta _{EV}$ is centred around $32 {\pm }18$° (Fig. 2f). In contrast to the horizontal plane, it does not matter whether the fly turns left or right, the mean $\theta _{EV}$ always stays at 32° elevation. At first glance, it is impossible to know which parameter of $\theta _{AV}$ or $\theta _{EV}$ the fly is trying to keep constant. Thus, the fly may use in elevation a constant target heading angle controller (Eq. (1.2) with $\beta = 32$°), or a constant bearing angle controller (Eq. (1.3)), or an hybrid controller (Eq. (1.4)). We will address this question in the next section.

### Kinematics: control of steering

**Figure 3:**
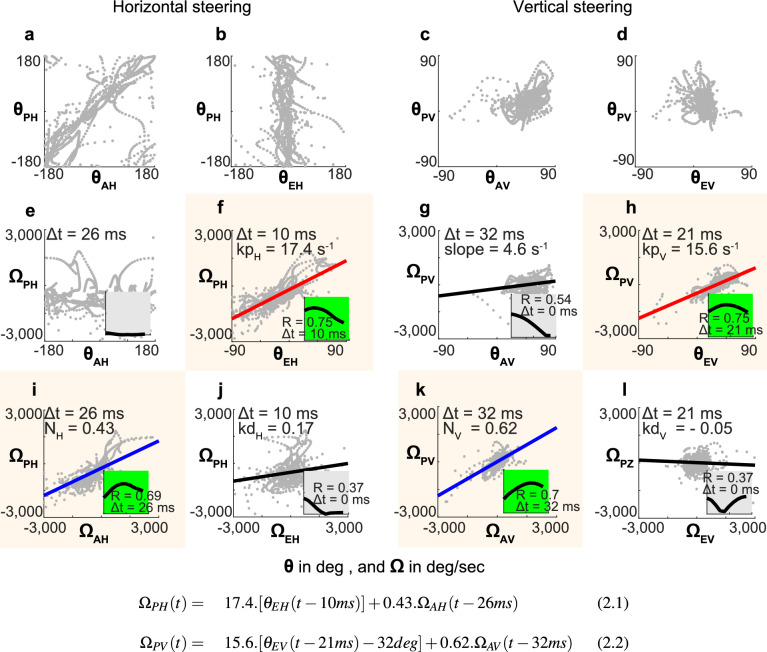
Impact of angular and angular rate parameters on the heading. (**a**–**d**) Heading angle $\theta _{P}$ as a function of absolute bearing angle $\theta _{A}$ and bearing angle $\theta _{E}$. While in azimuth, the angular range covers ${\pm }$ 180°, in elevation angular range is ${\pm }$ 90°. (**e**–**h**) Change of heading rate $\Omega _{P}$ as a function of angles $\theta _{A}$ and $\theta _{E}$. (**i**–**l**) Change of heading rate $\Omega _{P}$ as a function of angular velocity $\Omega _{A}$ and $\Omega _{E}$. Maximum correlation *R* and its delay $\Delta t$ are displayed in green insets if $R \ge 0.7$. Scale *X* = [0:50 ms], *Y* = [0:1]. Red lines show linear fits between $\theta _E$ and $\Omega _P$ suggesting a biased pursuit strategy. Blue lines show the linear fits between $\Omega _A$ and $\Omega _P$ indicative of a proportional navigation strategy. Gains (*kp* and *N*) and delays ($\Delta t$), from coloured graphs were used in the two control Eqs. (2.1) and (2.2). To facilitate comparison of linear fits between $\theta _{E}$ and $\Omega _P$ in horizontal and vertical planes in (**f**) and (**h**), respectively, the same angular range of ${\pm }$ 90° is applied for $\theta _{EH}$ and $\theta _{EV}$. $93\%$ of all $\theta _{EH}$ were in this angular range.

We began by looking at the relationship between $\theta _P$, $\theta _A$ and $\theta _E$. In the horizontal plane, $\theta _P$ = $\theta _A$ (Fig. 3a), whereas $\theta _E$ is maintained around 0° (Fig. 3b). It confirms the hypothesis that the fly tries to stabilize $\theta _E$. In the vertical plane, the values of $\theta _P$, $\theta _A$ and $\theta _E$ stay more or less constant (Fig. 3c,d). For further investigations we need to introduce the angular velocities $\Omega _P$, $\Omega _A$ and $\Omega _E$, which correspond to the first temporal derivatives of $\theta _P$, $\theta _A$ and $\theta _E$, respectively.

#### Horizontal plane: hybrid control for tracking

Essentially, the change of steering, $\Omega _P$, should be strongly correlated with $\theta _E$ and $\Omega _E$ if the pursuer follows a *PP* or a *BP* strategy (Eqs. (1.1, 1.2)), or with $\Omega _A$ if it follows a *PN* strategy (Eq. (1.3)). The analysis of our data reveals that $\Omega _P$ has a strong linear correlation with $\theta _E$ (*R* = 0.75) and with $\Omega _A$ (*R* = 0.7). The maximum correlation (*R* = 0.75) is for $\Omega _P = kp \cdot \theta _E (t-\Delta t)$ with *kp* = 17.4 $\hbox {s}^{-1}$ and $\Delta t$ = 10 ms (Fig. 3f). We found a very low correlation between $\Omega _P$ and $\Omega _E$ (Fig. 3j). As the fly employs only a proportional controller—and not a proportional derivative—, the 24Hz modulations of target’s velocity have been filtered out, thus they don’t have any impact on the pursuer’s steering (for details about this 24 Hz modulation see “Methods”). Most aerial chasing insects which employ a *PP* use a proportional-derivative controller to stabilise $\Omega _P$. This includes *Fania*^14^ as well as honeybee when tracking small moving platforms^25^. Dilochopodid flies, on the other hand, use a simple proportional controller^38^.

As if the fly followed a *PN* strategy, we found a good correlation (*R* = 0.7) between the variation of the bearing angle and the horizontal steering $\Omega _P = N \cdot \Omega _A (t-\Delta t)$, with *N* = 0.43 and $\Delta t$ = 26 ms (Fig. 3i).

Our analysis suggests that *Lucilia sericata* uses a hybrid steering control (Eq. (1.4)), similar to what has been observed in hawks^1^.

#### Vertical plane: hybrid control for interception

For steering in the vertical plane we found that the change of course, $\Omega _P$, is linked to the same parameters as for the horizontal plane. $\Omega _P$ is linearly related to $\theta _E$ (*R* = 0.75) with *kp* = 15.6 $\hbox {s}^{-1}$ and $\Delta t$ = 21 ms (Fig. 3h). $\Omega _P$ is also linearly related and to $\Omega _A$ (*R* = 0.7) with *N*= 0.62 and $\Delta t$ = 32 ms (Fig. 3k). Other similarity with the results found for the horizontal plane is that $\Omega _P$ in not linearly correlated with $\Omega _E$ (Fig. 3l). On the other hand, the curve $\Omega _P = k \cdot \theta _A$ in Fig. 3g has a non-negligible *R* of 0.5 that was not observed in horizontal plane (Fig. 3e). Because this maximum correlation was found for a zero delay between $\Omega _P$ and $\theta _A$, we have not included $\theta _A$ in the formulation of the control laws.

#### Similarities in the two planes of approach

There are conspicuous similarities between the coefficients we obtained for the equations describing the horizontal and vertical control: the data shown in Fig. 3f,h have the same profile which is also true for Fig. 3i,k: $kp_H = 17.4 \,\text{s}^{-1}$, $kp_V = 15.6 \,\text{s}^{-1}$, $N_H = 0.43$, and $N_V = 0.62$. However the differences are notable on the sensorimotor delays. For vertical corrections between $\theta _ E$ and $\Omega _P$, the delay is twice as long as the one for horizontal corrections ($\Delta t_H$ = 10 ms and $\Delta t_V$ = 21 ms). The delay is also longer for vertical corrections between $\Omega _A$ and $\Omega _P$ ($\Delta t_H$ = 26 ms and $\Delta t_V$ = 32 ms). We have already shown that in the vertical dimension variances of angular parameters are smaller than for the horizontal dimension.

The correlations between $\Omega _P$ and kinematic-related parameters ($\theta _E$, $\theta _A$, $\Omega _E$ and $\Omega _A$) give rise to useful observational relationships. It becomes important to consider building a model to understand the contribution of each relationship to the global steering strategy.

### Kinematics: control of speed

**Figure 4:**
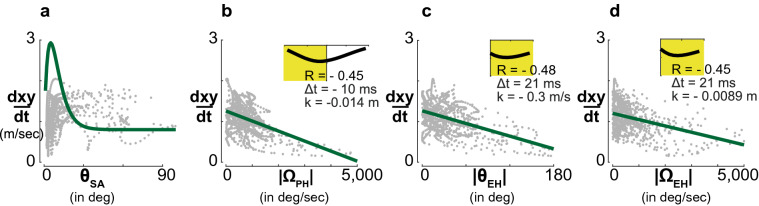
(**a**) Relationship between pursuer horizontal speed and target angular size. Measured data (gray dots) and control law (green curve) Boeddeker et al.^17^ proposed as described in Eq. (3). Our data are not described well by the proposed control law. (**b**) Relationship between pursuer horizontal speed and its angular velocity. Fast angular rotations are (weakly) correlated with a lower translational speed, as described for coordinated turns. Cross correlation analysis shows that the best linear fit is observed when deceleration occurs 10 ms before the turn. (**c**) Relationship between the horizontal speed and the horizontal target heading angle $\theta _{EH}$ (**d**) Relationship between the horizontal speed and the horizontal target heading angle rate $\Omega _{EH}$. In (**b**–**d**) relationships between horizontal speed and angular parameters show a weak linear correlation $R < 0.5$.

The modeling of the chasing strategies are sometimes limited to the characterisation of 2D or 3D steering without much consideration about forward speed control. Boeddeker et al.^17^ developed a virtual blowfly to model chasing behaviour. They implemented a speed controller based on the apparent angular size of the target (see Eq. 3). With this controller, the authors were able to include the phenomenon that some flies got stuck at a certain distance from the target, which they called: *Pursuit* chases. Getting closer to the target creates an image expansion of the target triggering deceleration, while image contraction due to an increased distance initiates acceleration. The underlying relationship between the target size and forward speed is given by the curve shown in Fig. 4a. There is a conspicuous difference between the curve presented by Boeddeker^17^ and our experimental data. The most substantial differences are along the shape of the curve on the one hand and the distribution of our data on the other. These discrepancies are possibly due to different turning radii of the dummy trajectories used in the two studies. Boeddeker^17^ applied a larger turning radius than we did in our experiments, which enabled the flies to reach higher forward speeds. So we looked for an alternative control law for forward speed and found an average linear correlation of R $\sim$ − 0.5 between horizontal speed and $\theta _{EH}$, which was the same between horizontal speed and $\Omega _{EH}$ (see Fig. 4c,d).

3 $$s\left(t\right)=\left\{\begin{array}{cl}
{S_{\mathrm{g}}} & {\text { if } \rho \leqslant 0.5^{\circ}} \\
{\rho\left(t-\Delta t\right) S_{\mathrm{v}} e^{-\rho\left(t-
\Delta t\right) / \rho^{*}}+S_{\mathrm{g}}} & {\text { if }
\rho>0.5^{\circ}}
\end{array}\right.$$

### Developing kinematic models

In a first step, we simulated the responses of a virtual fly by implementing the steering control Eqs. (2.1, 2.2) in Matlab/Simulink 2019. We used the experimental data to specify the initial conditions and forward speed used in our simulations. The implementation of a forward speed controller, based on the relationships presented in Fig. 4, did not give satisfactory results, which is probably explained by their low correlation coefficients between horizontal speed and the values of $\theta _{EH}$ $\Omega _{EH}$. Thus, the speed of the model fly is set to be equal to the speed of the real fly—i.e it changes from moment to moment, depending upon the instantaneous speed of the real fly.

The trajectories of the simulated fly were evaluated based on their deviation from the trajectory of the experimental animal by the error, $\varepsilon$, defined as the mean absolute distance between the measured ($\hat{x}_P,\hat{y}_P,\hat{z}_P$) and simulated ($x_P,y_P,z_P$) positions of the pursuer at each time point:

4 $$\varepsilon _{H}=\frac{1}{n} \sum _{k=1}^{(n)}\sqrt{{(\hat{x}_{P(n)}-x_{P(n)})}^2 + {(\hat{y}_{P(n)}-y_{P(n)})}^2}$$

5 $$\varepsilon _{V}=\frac{1}{n} \sum _{k=1}^{(n)}\sqrt{(\hat{x'}_{P(n)}-x'_{P(n)})^2 + (\hat{z}_{P(n)}-z_{P(n)})^2}$$

where *x′* corresponds to the horizontal displacement, see Eq. (9).

Based on the model derived from behavioural parameters, we created three virtual fly models, and tested them both for the horizontal and the vertical plane. The models simulated: (*i*) biased pursuit, *BP*, (*ii*) proportional navigation, *PN* and (*iii*) a mixed pursuit strategy, *MP*, which combines biased pursuit and proportional navigation. The gains implemented in each model were estimated using the smallest error, $\varepsilon$, as a performance measure.

**Figure 5:**
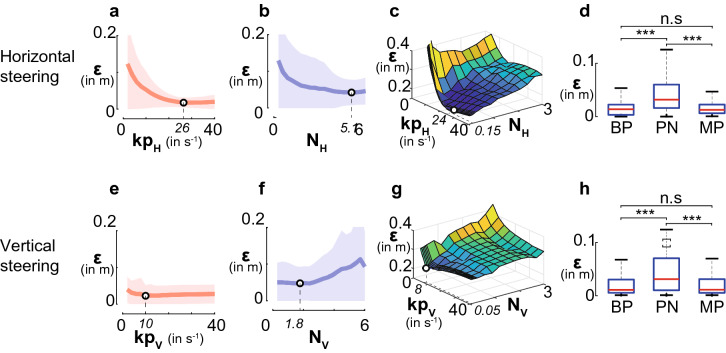
Optimization of gains for the three steering control models. Reduction of the error between model and real trajectories, were quantified by the error $\varepsilon$ according to Eqs. (4, 5). (**a**,**e**) Biased pursuit, *BP*, minimum $\varepsilon$ for $kp_H$ = 26 $\text{s}^{-1}$ and $kp_V$ = 10 $\text{s}^{-1}$. (**b**,**f**) Proportional navigation, *PN*, minimum error $\varepsilon$ obtained for $N_H$ = 5.1 and $N_V$ = 1.8. (**c**,**g**) Mixed pursuit, *MP*, minimum $\varepsilon$ obtained by varying *kp* and *N*. Horizontally $kp_H$ = 24 $\text{s}^{-1}$ and $N_H$ = 0.15, and vertically $kp_V$ = 8 $\text{s}^{-1}$ and $N_V$ = 0.05. The thick lines in (**a**,**b**,**e**,**f**) represent the average $\varepsilon$ obtained over all 17 captures. Shaded areas indicate standard deviations. (**d**,**h**) Box plot of all errors $\varepsilon$ when using models with parameters values from (**a**–**c**,**e**–**g**), and *ANOVA* tests. There is no significant difference between the *BP* and *MP* strategies in horizontal and vertical direction (n.s: *p* $> 0.05$). Note that the *PN* strategy induces bigger error compared to the two others. (***p $< 0.001$).

**Figure 6:**
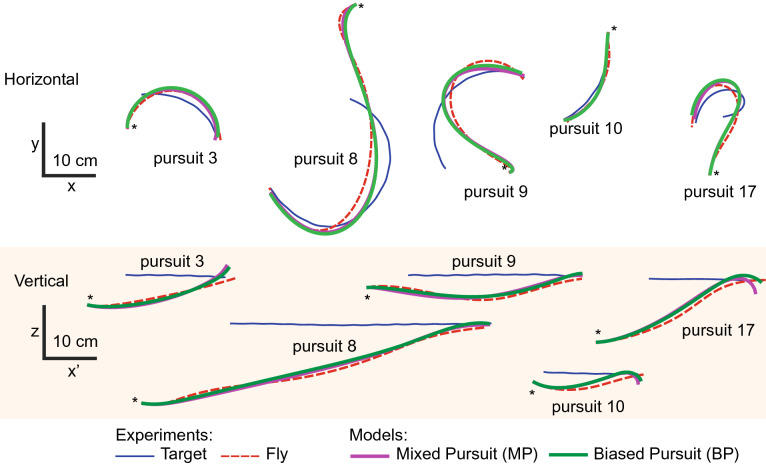
Experimental and model trajectories. Five of the 17 studied chases are plotted and compared to the trajectories of a model fly whose steering control is governed by mixed pursuit strategy, *MP*: Eq. (1.4) with parameters from Fig. 5**c**,**g**, and biased pursuit strategy, *BP*: Eq. (1.2) with *kp* from Fig. 5**a**,**e**. Upper and lower row of trajectories show projections in the horizontal and vertical plane, respectively. Asterisks indicate the starting point of the chases. The speed of the model fly was set to correspond to the speed of the real fly. For the vertical plane: x′ is the displacement along horizontal plane (see Eq. (9)). Note that the *MP* and *BP* models produce highly similar results which come close to the trajectories of the real fly. To quantify the differences between experimental and model trajectories we used a point-to-point error, $\varepsilon$ (Eqs. (4, 5)).

We then compared the performance of the different models to real pursuits. The *MP* and *BP* models performed best and second best, respectively, with the *PN* model coming third. We did not find a significant performance difference between the *MP* and the *BP* model, neither in the horizontal nor in the vertical plane (Figs. 5, 6).

The comparison of the model performances may suggest that *PN* has no sizable impact on the fly’s control strategy. On the other hand, if *PN* is not necessary, but we observed a linear relationship between $\Omega _P$ and $\Omega _A$, how can we exclude *PN*? One answer can come from the small value of the coefficient *N*. When *PN* strategies are applied in nature, *N* is always bigger than one (see “Introduction”). Here we found $N_H = 0.43$ and $N_V = 0.62$ for the behavioural data (see Fig. 3i,k), and $N_H = 0.15$ and $N_V = 0.05$ for the *MP* model (see Fig. 5c,g). The advantages of such a small *N* coefficient are rather unclear even if the *PN* and *BP* strategies are combined. Overall, our results suggest that the control strategy offering the best performance is the biased pursuit with a proportional controller in both azimuth and elevation heading control. Finally, we varied the bias angle values in our *BP* model for elevation, and found that $\beta = 23$° gave the best performances.

6 $$\begin{aligned}&\Omega _{PH} (t) = 26 \cdot [ \theta _{EH} (t - 10 \; \text{ms}) ] \end{aligned}$$

7 $$\begin{aligned}&\Omega _{PV} (t) = 10 \cdot [ \theta _{EV} (t - 21 \; \text{ms}) - 23^{\circ}] \end{aligned}$$

The difference in strategy between the two planes lies essentially in the value of the bias angle, $\beta$, gain, *kp*, and the time delay $\Delta t$. In the horizontal plane $\beta _{H} = 0$°, which leads to a tracking strategy. In the vertical plane $\beta _{V} = 23$° which leads to an interception strategy. Hypotheses concerning the use of the two pursuit strategies, tracking along azimuth and interception along elevation, will be developed in the next section.

## Discussion

The two strategies observed, horizontal tracking and vertical interception, would therefore require to maintain a different constant target heading angle $\theta _E$ depending on the plane of approach. The horizontal angle of error, $\theta _{EH}$ tends to be equal to zero since the body axis is usually aligned with the speed vector. In some cases, however, the body axis and speed vector are not aligned, for instance during side-slip—as a result of inertia during high speed banked turns, acrobatic moves during chasing flights^37^, or—more importantly—after sudden body saccades (rotations around the vertical axis)^39^.

In the vertical plane, this misalignment between speed vector and line of sight, LOS, may also be explained by the location of the acute zones in the fronto dorsal part of the compound eye of male dipteran flies^40^ (described in “Introduction” for dragonflies). This area features larger lenses that capture more light, increasing light sensitivity, faster photoreceptor responses, and neural connections feeding into sex specific pathways^36,41,42^. These sexual dimorphisms support male chasing behaviour and have probably developed under high evolutionary pressure. In the vertical plane, however, we know that the body axis is hardly aligned with the speed vector, nor with the LOS^37^. The role that the orientation of the body plays in the dynamics of the pursuit is the subject of an article in preparation.

Unlike male blowflies, dragonflies use interception strategies in both azimuth and elevation. Why would the fly change the strategy of its successful ancestors, in addition to completely different flight aerodynamics and therefore a different control ability? One answer could come from the movements of the head. During pursuit, the dragonfly head moves to stay locked to the target^28,43^. The position of the target is maintained in the acute zone. Even *Drosophila*, who does not possess an acute zone presents head movements in the context of tracking and compensation of background motion^44^. Contrary to the high neck mobility of dragonflies, blowflies can only rotate their head with a maximum head-thorax yaw angle of ${\pm }$ 20°^45^, and a 10° mean peak of head-thorax yaw angle during saccades^46^. In our experiment, the data were normalized to a target rotating in an anticlockwise direction. This created an offset for the target heading angle, $\theta _{EH}$ = 21°, as presented in Fig. 2c (more details are discussed in^37^). The $\theta _{EH}$ offset, and the maximum head-thorax yaw angle measured in blowflies are very similar. To define their relationship one requires head-body angle measurement, which would be a challenge to determine during pursuit.

The extremely fast control of horizontal heading direction of 10 ms ensures a tight visual connection with the target, which may partially compensate for the low mobility of the neck in the case of horizontal tracking. The same argument would hold for the vertical pursuit, as the angular range for pitch head movements in Calliphora is also just ${\pm }$ 20°^45^. So why is vertical strategy different than the horizontal one? As opposed to pure pursuit, interception is more energy-efficient^47^. If the fly employed a pure pursuit strategy in the vertical plane it would take the risk of overshooting, causing considerable energy losses, the more so as it would have to fight gravity. After all, chasing is energy-intense and may be used as fitness selection criterion. Only the fittest (in terms of sensory processing/accuracy and flight performance) males get to mate and produce offspring.

To capture the target the pursuer can follow different pursuit strategies as described in “Introduction”, i.e tracking or interception. The resulting trajectories may be implemented by smooth continuous (smooth pursuit) or almost step-like (steering-saccade) functions. The use of body-saccades plays an important role in stabilising the gaze^48^ during locomotion. Translation generates wide field retinal image shifts, or optic flow fields, containing relative distance information based on motion parallax. This information, however, is masked by distance-independent rotational optic flow which is of higher magnitude and relevant for flight stability and gaze control. To minimise the time during which other visual information is masked by rotational optic flow, *Calliphora* is known to perform fast gaze shifts in form of head- and body- saccades during cruising flight^49,50^.

To find out whether *Lucilia* performed body-saccades during its chasing flights we extracted angular rotation peaks—which reached values of up to 7000°/s—and associated changes in forward speed from our free flight data. We isolated the 6 fastest yaw rotations and yaw speeds higher than 1500°/s. The analysis of these segments demonstrated an expected reduction of the forward speed when the fly performed those spectacular saccadic rotations (Supplementary Figure S2). Deceleration of the forward speed, coupled with high yaw rotation describes a maneuver called coordinated turns, in which the fly controls its centripetal acceleration to avoid side-slips. This maneuver has been described in loitering honeybees^51^.

To study steering saccades, Braun et al.^50^ extracted free ‘flight primitives’ (or prototypical movements) by cluster analysis of flight features such as translational speeds or angular velocities. Researchers applied this method on cruising flight in blowfly and hoverfly^52,53^. They quantified the differences of flight attitude between the two species. It would be very interesting to use this technique on our pursuit flight data to compare the prototypical movements of the male blowfly chasing and cruising. For now, we analyzed the relationship between forward speed and yaw angular velocity in horizontal plane, and we found that the forward speed decreases 10 ms before the onset of the yaw rotation (Fig. 4b), which is in line with what was described in houseflies^14^.

As the z-position (altitude) of the target was not varied in our experiments, the dynamic input range we used to identify the vertical control strategy is somewhat limited. Incidentally, it is also in the vertical dimension where the previously cited hypothesis presented by Strydom^47^ appears not to apply. In the vertical plane, *kd*/*kp* = 3 ms which is different from the delay of 26 ms measured in our experiments. Further experiments where the z-position is systematically varied would help to overcome the current limitation.

Although the vertical input range is limited due to the constant *z*-position of the dummy, different initial conditions regarding the start positions for the flies’ chasing flights introduce a certain degree of variability in altitude-related parameters. To our knowledge, the rare studies on vertical approach have been realised with targets moving in the horizontal plane for dragonfly^43^, robberfly^30^ and killerfly^34^. In another experiment with drone bees pursuing a suspended queen, van Praagh et al.^54^ measured the elevation angle between body axis and line of sight. Distribution of this angle (noted $\alpha$) is similar to our measures of $\theta _{EV}$ and $\theta _{AV}$. It is reassuring that our flies keep the target projected onto a vertical angular range which corresponds well with the position of the acute zone, such as described for the drone bee. Indeed blowfly males and drone bees (also males) share several morphological properties such as body size, restricted movements of the head during flight, and the presence of a dorsal acute zone.

Different pursuit strategies in the horizontal and vertical plane as suggested by our study may be, at least partly, the result of the fly’s specific body shape. The asymmetric mass distribution and shape along the yaw and pitch axes are likely to be differentially affected by the inertial vector and gravity. This may impose different dynamics for horizontal and vertical steering which could have facilitated the development of the separate pursuit strategies.

Although only at a low coefficient of $R = 0.5$, there is a correlation between $\Omega _{PH}$ and $\Omega _{PV}$. Indeed we found a linear relation of the form: $\Omega _{PZ(t)} = k \cdot \Omega _{PH(t-\Delta t)} + A$ with $k = -0.25$, $\Delta t = 21$ ms, and A = 3.85 $\text{s}^{-1}$ (see Supplementary Figure S3). A large path change in horizontal plane—independent of direction of rotation—is followed by a negative vertical rotation, i.e. a downward rotation. This phenomenon can be explained by the presence of banked turns.

In fixed wing aircraft, changes in heading direction is usually performed with banked turns, such as in fruit flies^55^, or in bioinspired flapping wing aerial robot^56^. To turn left the body rolls along its longitudinal axis to the left. The projection of the lift following this rotation onto the horizontal plane is a force orthogonal to the speed vector creating a change of heading, that is inversely proportional to the forward speed (Equation (1) in Supplementary Figure S2). The gain in force due to the yaw rotation is compensated by negative vertical lift resulting in a loss of altitude. However, when the relationship between horizontal and vertical steering is implemented in the vertical steering control, we did not observe any significant improvement of its performance. To confirm the presence of banked turns in blowfly pursuit, roll-angle measurements would be required, which is a technical challenge in free flight experiments.

In summary, we have analyzed a series of 17 chasing flights where a male blowfly was pursuing a moving dummy. The analysis of the resulting trajectories suggests that the pursuit strategy is not the same along the 3 dimensions. Our comparative modelling study provides evidence that a constant target heading angle controller best captures the kinematics of the chasing flights we have analyzed. This controller leads to tracking if driven by the target heading angle in the horizontal plane, and it leads to interception if driven by a biased elevation angle in the vertical plane. Thus we can assume that constant target heading angle is the general strategy and both *tracking* and *interception* are just consequences of the presence of the bias angle. The difference between tracking in the horizontal plane and interception in the vertical plane may be explained by a trade off between evolutionary fitness and energy efficiency, respectively, but may require further studies to support this interpretation. It is both beautiful and remarkable that the combination of two simple proportional controllers are capable of reproducing behaviour as complex as fly chasing flights at ultra-fast time scales. This is in line with the Braitenberg vehicles’s^57^ spirit where the synthesis is often simpler than the analysis. Such parsimonious design may be a source of inspiration when it comes to the development of novel control architectures for aerial robotic platforms.

## Methods

**Figure 7:**
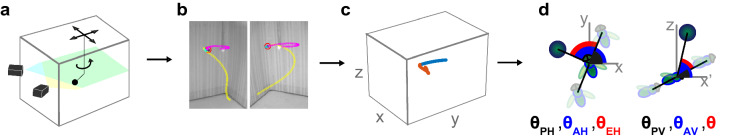
Road map of the kinematic study of blowfly aerial pursuit in 3D. (**a**) Schematic view of the chasing arena, the moving target and the two cameras with overlapping fields of view. (**b**) Two synchronised frames and the 2D tracking of target and fly with toolbox *DLTdv5*^58^. (**c**) 3D reconstruction of the target and the pursuer positions, in blue and red, respectively, using Matlab2018, *DLTdv5*^58^ and a dedicated toolkit^37^. (**d**) Graphical definition of the angular parameters in horizontal and vertical planes. Heading angle, bearing angle, and error angle, $\theta _P, \theta _A$ and $\theta _E$, respectively, as defined in Eq. (8). We distinguished values in the horizontal plane (noted *H*) and in the vertical plane (noted *V*), where angles are measured relative to *x*′-axis, the horizontal displacement defined in Eq. (9).

### Animals

We used a recently developed setup^37^ to record chasing flights in blowflies (*Lucilia sericata*). Pupae were purchased from an animal supplier (BioFlyTech) in Spain. Male flies aged between 5 and 12 days were placed in the arena. They were exposed to a 12:12 h light:dark cycle with a luminance of about 2000 cd $\text {m}^{-2}$ at a temperature between 20 and 25 °C. 20 males stayed in the arena without engaging in an experiment for one day to get used to their new environment. The experiments were recorded during the 5 consecutive days around noon, during the high diurnal activity phase. Every day we presented to the flies 20 repetitions of each target trajectories, with 1 min between each experiment.

### Videography

The volume of the chasing flight arena (50 × 50 × 70 cm) was almost entirely observed by two stereovision cameras (PROSILICA GC640) with a spatial resolution of 640x480 pixels. A schematic view of the arena and the two stereovision cameras is presented in Fig. 7a. We recorded at a temporal resolution of 190 frames per second. The cameras were equipped with optics used at fixed focal depth (6 mm, F = 1.4).

The synchronised images from both cameras were analysed and the 2D positions of the fly and the target were obtained using the toolbox DLTdv5 developed by Hedrick’s lab^58^ (see Fig. 7b for an example of 2D object tracking using this toolbox). Its 3D reconstruction tool was then applied to obtain the spatial positions of the two protagonists (Fig. 7c) with a standard deviation of 5 mm and a time resolution of 5, 3 ms. A detailed description of the system has been previously published by Varennes et al.^37^.

### The dummy

#### Moving the target

The target was a small dark sphere of 8 mm diameter, similar to a female profile, which followed predefined trajectories in the flight arena. It moved on three degrees of freedom, two translations in the horizontal plane and one rotation around the vertical axis (see^37^ for further details). These movements were controlled by three motors: two steppers and one DC motor. They were positioned outside the arena to reduce potential distractions of the flies. A system of belts and pulleys allowed the target to be positioned with an average error at each position of less than 5 mm along a course of 3 m long.

#### Target trajectories

Males were presented with two types of target trajectories for this study. In the first case, the target was moved on a circular path at a speed of 1 m/s. The second trajectory combined a translation along the y-axis with a rotation around the vertical axis, which created a spring-shaped movement of the target. The forward speed of the target varied between 0 and 1.5 m/s , and its angular velocity ranged between 360 and 1300°/s (while the rotation around the z-axis was kept constant). With these two trajectories, we presented to the pursuers a variety of dummy kinematics, varied enough to study the sensorimotor control of the animal during its pursuit flight. Positions and velocities of purser and target are presented in Supplementary Figure S1. During circular trajectories, 24 Hz modulations of unknown origin appeared in the target’s translational velocity profile, as discussed later, they have been filtered out by the fly.

### Data analysis

#### Pursuit sequences

A fly was considered to engage on a pursuit flight when abruptly changing its speed and orienting towards the dummy. Pursuit flights normally ended by the fly catching the target. Flight trajectories were varied in shape, and distributed throughout the volume of the flight arena. The broad range of flight speeds we observed were in line with data reported for the slightly bigger blowfly species *Calliphora*^39,59^. General features of the chasing behaviour were comparable with results obtained in a previous study on *Lucilia*^60^. In our analysis, we only included flights with a final capture. Indeed, about $30 \%$ of the chases were abandoned in flight. This figure is roughly aligned with abandoned pursuits ratio of $43 \%$ in the muscoid fly *Coenosia* and $36\%$ in the asilid fly *Holcocephala*^34^. We were unable to identify the reason for the animals to abandon their chasing flights.

#### Variables of interest

To quantify the pursuit strategies observed in *Lucilia* we introduced the following parameters: line of sight, *LOS*, is the line connecting the centre of mass of the pursuer $(x_{P}, y_{P}, z_{P})$ to the centre of mass of the target $(x_{T}, y_{T}, z_{T})$. Line of flight, *LOF*, is the line connecting two consecutive positions of the pursuer, which is equivalent to its speed vector. The Cartesian coordinates of the positions were transformed into spherical coordinates. For the sake of clarity, spherical coordinates about *LOF* will be noted $(\theta _{PH}, \theta _{PV}, R_P)$, with *P* referring to the *pursuer*, and spherical coordinates relative to the *LOS* will be noted $(\theta _{AH}, \theta _{AV}, R_A)$ with *A* referring to *absolute bearing angle*. The spherical radii *R* represent the distance to the target for the *LOS* noted $R_A$, and the distance travelled by the fly per time unit for the *LOF* noted $R_P$.

*LOF* and *LOS* forms with the absolute reference frame an azimuth angle $\theta _{H} \, \in \, [-180^{\circ}{:}180^{\circ}]$ deg and an elevation angle $\theta _V \, \in \, [-90^{\circ}{:}90^{\circ}]$ deg. Finally the target heading angle or *error angle* noted $\theta _E$ is also composed of a horizontal and a vertical component. They are formed between LOS and LOF, in other words the difference between $\theta _A$ and $\theta _P$.

8 $$\begin{aligned} \left( {\begin{array}{c}\theta _{EH}\\ \theta _{EV}\end{array}}\right) =\left( {\begin{array}{c}\theta _{AH}\\ \theta _{AV}\end{array}}\right) -\left( {\begin{array}{c}\theta _{PH}\\ \theta _{PV}\end{array}}\right) \end{aligned}$$

When we present the vertical plan of the pursuit (Fig. 7d *right*) we plot the elevation on the *y*-axis against the absolute horizontal displacement, *x*′, on the *x*-axis.

9 $$\begin{aligned} \begin{aligned} x'_{P\textit{(n)}} = \sum _{k=1}^{n} \left | \sqrt{(\hat{x}_{P\textit{(n+1)}}-x_{P\textit{(n+1)}})^2 + (\hat{y}_{P\textit{(n+1)}}-y_{P\textit{(n+1)}})^2} -\sqrt{(\hat{x}_{P\textit{(n)}}-x_{P\textit{(n)}})^2 + (\hat{y}_{P\textit{(n)}}-y_{P\textit{(n)}})^2} \right | \end{aligned} \end{aligned}$$

where ($\hat{x}_P,\hat{y}_P,\hat{z}_P$) are the measured and ($x_P,y_P,z_P$) are the simulated positions of the pursuer at each time point *n*.

## Supplementary information

Supplementary Information 1.

Supplementary Information 2.

## Data Availability

High-speed videos of the pursuits will be uploaded in supplementary data. Reconstructed trajectory data analysed during this study, and the matlab/ simulink models of the steering controllers are available in the following GitHub: https://github.com/veandre/blowfly-pursuit.
