# Supplementary material for: Two pursuit strategies for a single sensorimotor control task in blowfly
Source: Sci Rep. 2020 Nov 27;10:20762. doi: 10.1038/s41598-020-77607-9 (PMC7695743; doi:10.1038/s41598-020-77607-9)
Supplement: Supplementary file 1 — Supplementary Information 1. [file 41598_2020_77607_MOESM1_ESM.pdf]

Two pursuit strategies for a single sensorimotor control task in blowfly.

Leandre Varennes[1,2], Holger Krapp[2], and Stephane Viollet[1,*]

[1]Aix Marseille Univ, CNRS, ISM, Marseille, France
[2]Imperial College, Department of Bioengineering, London, SW7 2AS, United-Kingdom
[*]stephane.viollet@univ-amu.fr

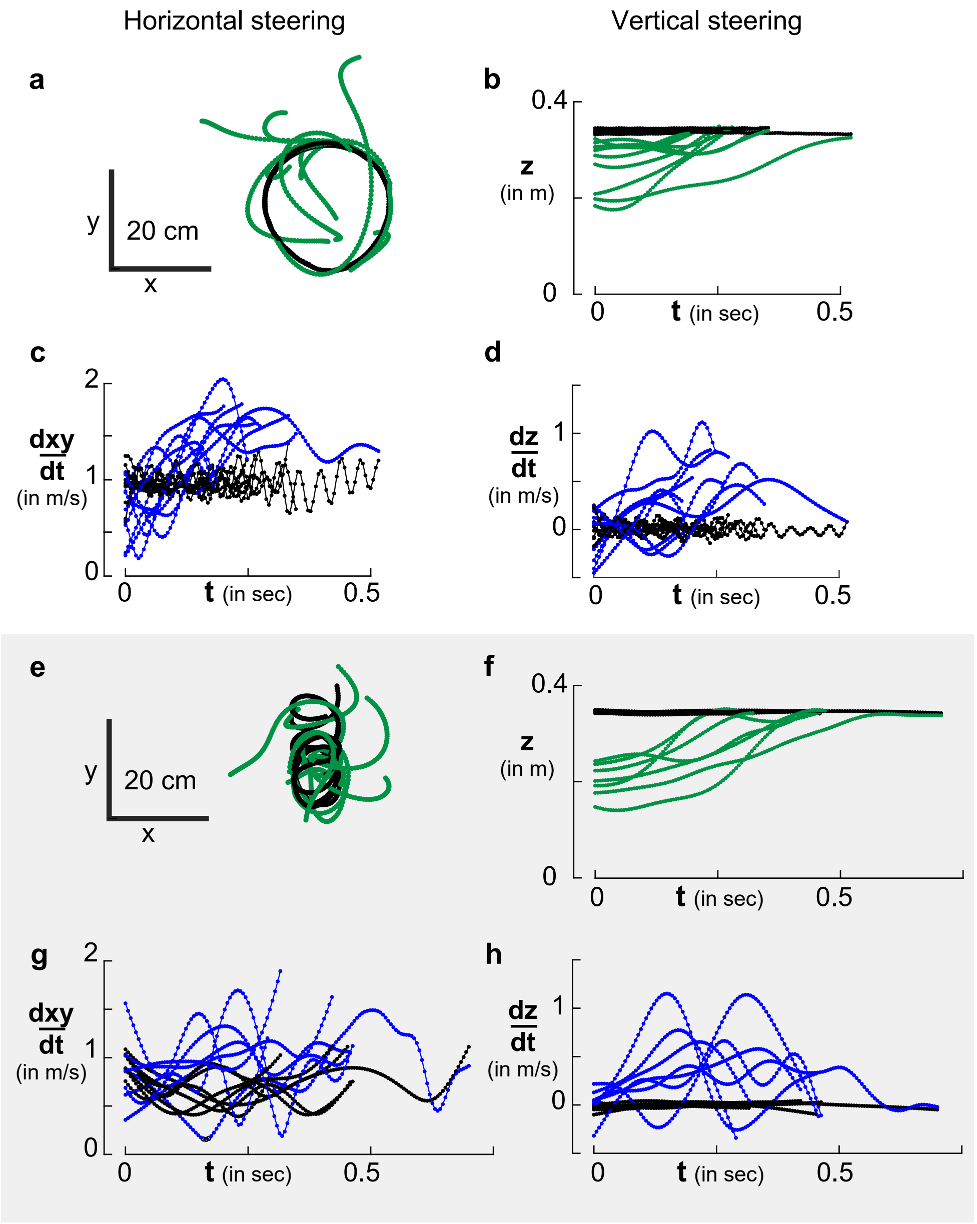

Supplementary Figure S 1. The 17 pursuit trajectories analyzed in this study. (**a-d**) The target moving on a circular trajectory, (**e-h**) the sping-shaped trajectory of the target. (**a, e**) show positions in the horizontal plane – defined by x- and y-coordinates – and (**b, f**) the vertical z-coordinate. The positions of the pursuer are in green, each point corresponds to a measurement, taken at 5ms time intervals. The horizontal and vertical speeds of the pursuer are shown in blue. Variations in the target's horizontal speed used to develop our kinematic fly model. Modulations of 24 Hz around 1m/s in circular trajectories are filtered out. We cannot conclude about 4Hz variations on eliptical trajectories. In the vertical plane, flies approach the target from below without ever overshooting the target's position.

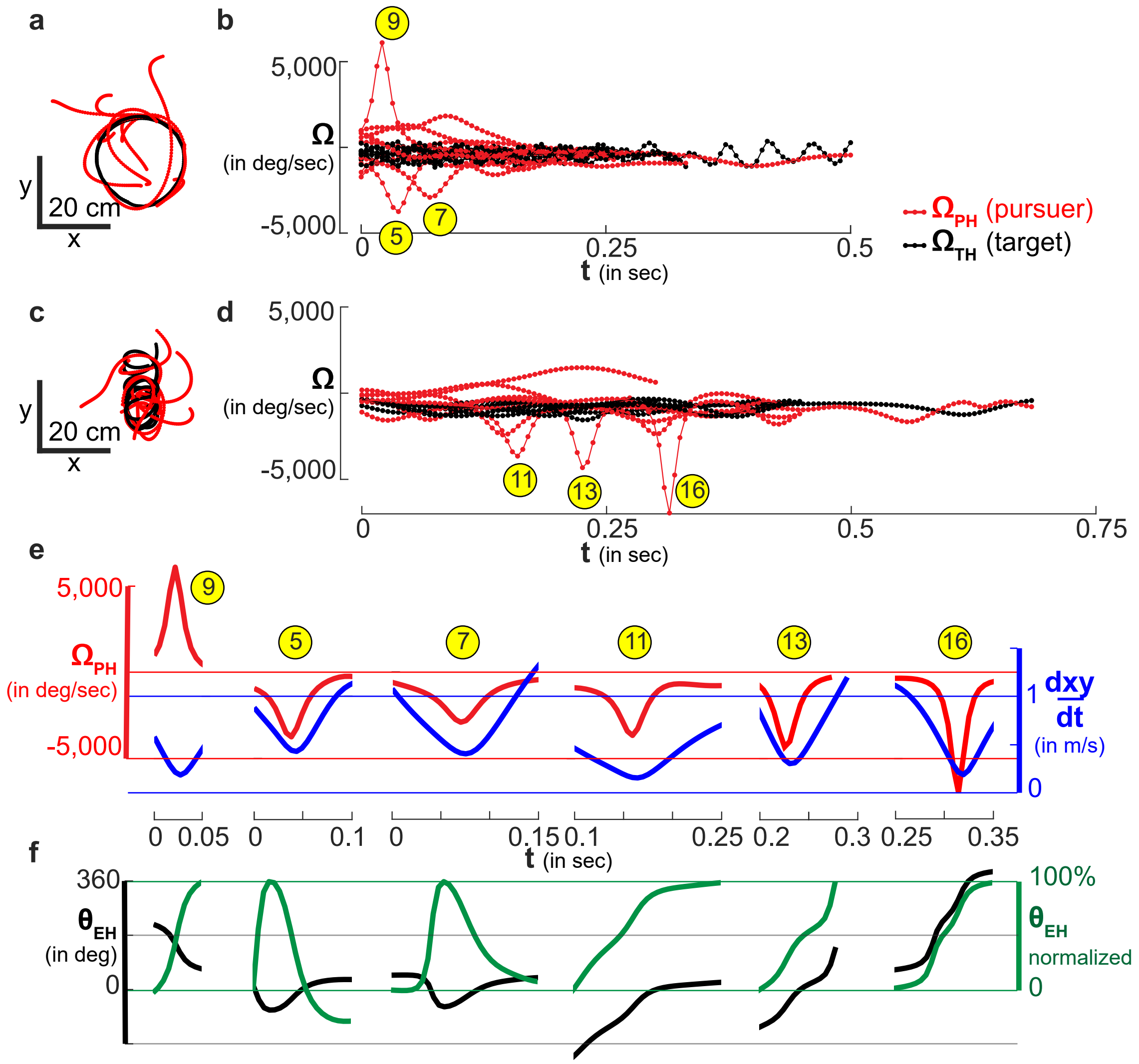

$$\Omega_P = \frac{g}{\frac{dxy}{dt}}.tan(\theta_{roll}) \quad (1)$$

Supplementary Figure S 2. Analysis of body- saccades. (**a**) Horizontal positions of target and pursuer, in black and red, respectively, when chasing the dummy moving on a circular path. (**b**) Angular velocity profiles. (**c**) Horizontal positions when when chasing the dummy moving on a spiral-shaped path. (**d**) Angular velocity profiles. (**e**) Simultaneous evolution of the rotation speed Ω_{PH} and forward speed, *dxy/dt*. Peaks are presented when $\Omega_{PH} > 1500$ deg/sec. The fly's forward speed decreases when the (absolute) angular velocity increases. (**f**) Evolution of the bearing angle while the fly performs this rapid body rotation. Turns 5 and 7 are catch up saccades trying to reduce the error angle, otherwise θ_E does not provide valuable information. Equation 1 describes banked turn with g the gravity acceleration, where a high rotation velocity can be achieved by reducing forward speed dxy/dt or increasing the roll angle θ_{roll}.

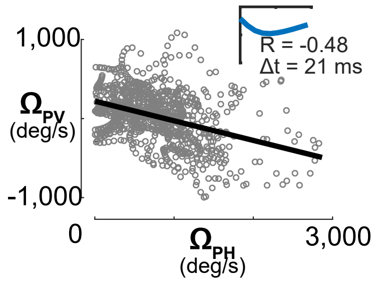

Supplementary Figure S 3. Relationship between horizontal and vertical steering. The cross correlation gives a weak maximum ($R = -0.48$) for a 21 ms delay. We can quantify the relationship by a linear regression (black line) of the form: $\Omega_{PV(t)} = -0.25.|\Omega_{PH(t-21ms)}|$ for $0 < |\Omega_{PH}| < 1500$ deg/sec
